# Oxidative Stress, Apoptosis, and Mitochondrial Function in Diabetic Nephropathy

**DOI:** 10.1155/2018/1875870

**Published:** 2018-04-01

**Authors:** Sonia Sifuentes-Franco, Diego Enrique Padilla-Tejeda, Sandra Carrillo-Ibarra, Alejandra Guillermina Miranda-Díaz

**Affiliations:** ^1^Institute of Experimental and Clinical Therapeutics, Department of Physiology, University Health Sciences Centre, University of Guadalajara, Guadalajara, JAL, Mexico; ^2^Programa de Químico Farmacéutico Biotecnologo, Escuela de Ciencias de la Salud, Campus Zapopan, Universidad del Valle de México, Guadalajara, JAL, Mexico

## Abstract

Diabetic nephropathy (DN) is the second most frequent and prevalent complication of diabetes mellitus (DM). The increase in the production of oxidative stress (OS) is induced by the persistent hyperglycemic state capable of producing oxidative damage to the macromolecules (lipids, carbohydrates, proteins, and nucleic acids). OS favors the production of oxidative damage to the histones of the double-chain DNA and affects expression of the DNA repairer enzyme which leads to cell death from apoptosis. The chronic hyperglycemic state unchains an increase in advanced glycation end-products (AGE) that interact through the cellular receptors to favor activation of the transcription factor NF-*κ*B and the protein kinase C (PKC) system, leading to the appearance of inflammation, growth, and augmentation of synthesis of the extracellular matrix (ECM) in DN. The reactive oxygen species (ROS) play an important role in the pathogenesis of diabetic complications because the production of ROS increases during the persistent hyperglycemia. The primary source of the excessive production of ROS is the mitochondria with the capacity to exceed production of endogenous antioxidants. Due to the fact that the mechanisms involved in the development of DN have not been fully clarified, there are different approaches to specific therapeutic targets or adjuvant management alternatives in the control of glycemia in DN.

## 1. Introduction

The global prevalence of diabetes mellitus (DM) in adults has increased considerably in recent decades. In the 7th edition of the diabetes mellitus (DM) Atlas published in 2015, it was reported that there are 415 million adults with DM in the world and it is estimated that there will be 693 million people living with DM by the year 2045 [1]. The lack of adequate control in consistently high levels of glucose leads to the appearance of serious micro- and macrovascular complications. The characteristic macrovascular complications include cardiovascular disease (CVD) with the propensity to suffer heart attacks and cerebrovascular accidents. Among the microvascular complications, neuropathy, retinopathy, and diabetic nephropathy (DN) stand out [2].

DN is characterized by the appearance of persistent clinical albuminuria (albumin excretion rate (AER) > 300 mg/24 h) and a reduction in the glomerular filtration rate (GFR) > 5 years in the absence of urinary tract infections, other kidney diseases, or cardiac insufficiency [3]. Proteinuria occurs in ~15–40% of type 1 DM patients and between 5 and 20% of individuals with type 2 DM. DN has significant long-term effects on the morbidity and mortality of DM patients [4]. Because the mechanisms in the appearance of DN have not yet been fully described, we proposed the approach of looking at diverse themes including the epidemiology, the mechanism of appearance, and the role of OS, the oxidative DNA damage, mitochondrial function, and cell death due to apoptosis in DN. Also described are some management alternatives that should be considered adjunctive in the control of glycemia and for the traditional DN risk factors. We emphasize that the best management for patients with DM to avoid and control the development of kidney damage is the adequate control of glycemia.

### 1.1. Epidemiology

Diabetes mellitus is the most common cause of chronic kidney disease (CKD) in the world, where approximately 1 out of every 4 adults with DM has CKD [5] and 1 of every 10–20% of patients with DM dies due to CKD [6]. According to a report from the United States Renal Data System (USRDS) in 2015, DM was identified as the primary cause of end-stage renal disease (ESRD) in >50% of patients treated in Singapore, Malaysia, Jalisco (Mexico), and Chile [7]. Diabetic nephropathy affects the African-American population about 3–5 times more than the Caucasian population. In type 1 DM, CKD is relatively rare in the first 5–10 years; however, the incidence rapidly increases in the following 10 years until reaching a maximum of about ~3% per year after suffering from DM for 15 years. After 15 years with type 1 DM, the percentage of CKD diminishes in 1% in ≥40 years with the illness. The patients suffering with type 1 DM for >35 years who have not yet developed DN have a low risk of presenting it [8].

In type 2 DM, DN develops predominantly in patients with systemic arterial hypertension and in the population with previous kidney disease. DN sometimes occurs in young individuals and can accompany diabetic retinopathy. In older persons with type 2 DM, retinopathy and proteinuria can be absent or minimal, although other kidney diseases should be investigated and excluded. Diabetic retinopathy and proteinuria could also be related to arterial hypertension [9].

### 1.2. The Development of Diabetic Nephropathy

DN is a condition of multiple stages that requires several years to arrive at being clinically evident. The development of microalbuminuria and the progression to manifested proteinuria are the common clinical characteristics of DN [10].

Microalbuminuria is the first indicator of DN, which can be detected at ~1 year from the onset of the type 1 DM. Microalbuminuria is already present now in the diagnosis of type 2 DM. At the onset of type 2 DM, structurally significant glomerular disease exists and the GFR is already beginning to be affected. In the case that no intervention was to occur, the microalbuminuria progresses to clinical albuminuria in ~10–15 years. Studies reported suggest that the natural history of DN is variable: in ~20%, microalbuminuria progresses to clinical albuminuria in 5–9 years, while in 50%, it remains in microalbuminuria. Microalbuminuria in ~30% of patients returns to normal ranges (<30 *μ*g/min). Microalbuminuria is a strong predictor of death by CVD in elderly patients with type 2 DM. Microalbuminuria is associated with generalized endothelial dysfunction in type 1 and type 2 DM. As proteinuria increases, the loss of GFR is quicker [9].

The natural history of DN differs between type 1 and type 2 DM. The five classic stages described in type 1 DM may not occur in type 2 DM because type 2 is diagnosed after other related but silent disturbances like arterial hypertension, proteinuria, or renal insufficiency. In type 1 DM, the alterations in kidney structure and function are found present at the onset of the illness. (A) Stage 1 is characterized by early hyperfunction and hypertrophy (changes are partially reversible with adequate treatment). (B) Stage 2 develops silently over the course of many years and is characterized by morphological lesions without clinical signs of illness. In this stage, good control of the DM does exist and the excretion of albumin is normalized. In deficient control of the hyperglycemic state, the excretion of albumin increases at rest and is greater during exercise. An important number of patients continue in stage 2 throughout their lives. (C) Stage 3 is characterized by incipient DN, which is the precursor to frank DN. Its main sign is the elevated urinary excretion of albumin. (D) Stage 4 is considered manifested DN. This is the classical entity characterized by persistent proteinuria (>0.5 g/24 h). When the arterial hypertension is not adequately treated, the GFR diminishes with an average fall of ~1 mL/min/month. (E) Stage 5 is considered ESRD with uremia [11]. In 2014, the group Committee on Diabetic Nephropathy reformulated the classification of DN [12] (Table 1).

**Figure 1:**
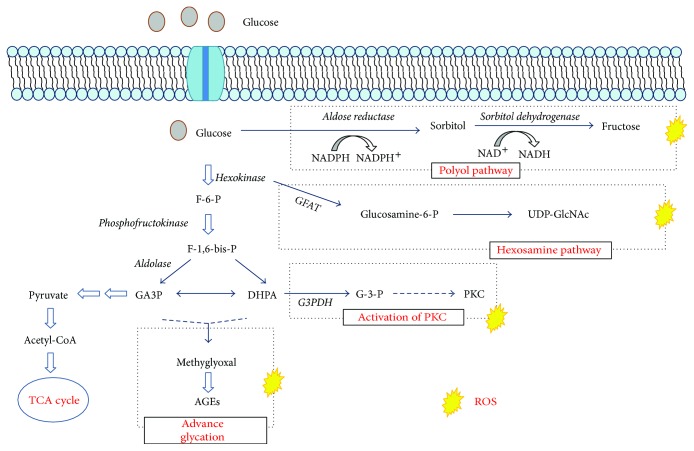
Mechanisms of cellular damage from the hyperglycemic state. Demonstration of the signaling pathways that are activated in the state of persistent hyperglycemia.

The development and progression of DN are influenced by diverse factors, among which the most important are hyperglycemia, arterial hypertension, obesity, and an unhealthy lifestyle [13]. Actually, sufficient evidence exists that indicates that oxidative stress (OS) is a factor of great importance in the development of type 1 and type 2 DM, generated primarily by the hyperglycemic state. Hyperglycemia causes tissue and endothelial damage through five primary mechanisms: (1) increase in the flow of glucose through the activation of the alternative metabolic pathways of glucose, the polyol pathway; (2) increase in the formation of intracellular advanced glycation end-products (AGE); increase in expression of the AGE receptor and activation of the ligands; (4) activation of isoforms of the protein kinase C (PKC); and (5) hyperactivity of the hexosamine pathway [14] (Figure 1).

The intracellular events induced in the presence of an environment of high concentrations of glucose favor the accentuated flow of polyols and hexosamine. The generation of AGE and reactive oxygen species (ROS), the activation of PKC, the activation of the transforming growth factor *β*-mitogen-activated Smad proteins (TGF-*β*-Smad-MAPK). Enzymes that degrade the extracellular matrix (ECM) have the ability to cause structural damage in the kidneys through the aberrant expression of cyclin-dependent kinases. When the AGEs are increased, they stimulate the glomerular cells to produce TGF-*β*1, which contributes to glomerular sclerosis and interstitial tubular damage through the abnormal production of ECM [15, 16]. The increase of ECM leads to kidney fibrosis primarily generated by the accumulation of mesangial cells, which favors the depositing of ECM, thickening of the glomerular and tubular membranes, dysfunction of the podocytes, and unchaining cell death by apoptosis. All of these events are caused by alterations in the redox system that leads to the appearance of albuminuria, proteinuria, glomerulosclerosis, and interstitial tubular fibrosis [17]. When the redox equilibrium is slightly altered, pathological processes are unchained that initiate producing damage to the macromolecules and that favor cellular dysfunction by contributing to the pathogenesis of DN [18]. By being the cellular nucleus, one of the primary objectives of the ROS is to generate structural changes that lead to cell death by apoptosis [19].

### 1.3. Oxidative Stress and Diabetic Nephropathy

OS is considered a common and important factor that links hyperglycemia with the vascular complications through metabolic changes to the molecules of the target tissues and the alterations in renal hemodynamics. Therefore, the metabolic attacks and hemodynamics from the increase in ROS have adverse synergic effects in the target tissues. Many ROS possess unpaired electrons and are considered free radicals. The ROS are a family of molecules that include molecular oxygen and its derivatives, among which are the superoxide anion (O^2−^), hydroxyl radical (HO^•^), hydrogen peroxide (H_2_O_2_), peroxynitrite (ONOO^−^), hypochlorous acid (HClO), nitric oxide (NO), and lipid radicals. Excessive quantities of ROS, after overcoming various endogenous mechanisms of antioxidant defense, oxidize various biomolecules like DNA, proteins, carbohydrates, and lipids, producing OS [20]. The potential sources of ROS include the mitochondrial respiratory chain, the xanthine oxidase, the NADH/NADPH oxidases, the NO synthase, and some other hemoproteins. In having divergent sources of ROS production, it is conceivable that different mechanisms operate in the generation of ROS in a hyperglycemic environment [21], because hyperglycemia favors the production of ROS. The ROS play an important role in the pathogenesis of diabetic complications [22]. The increase in the production of ROS can be so overwhelming, and the antioxidant defense systems are easily exhausted, favoring the increase in OS [23].

### 1.4. Mitochondrial Function and Diabetic Nephropathy

The ingested glucose generates energy for the production of adenosine triphosphate (ATP) through oxidative phosphorylation in the mitochondrial respiratory chain. After the glucose enters the cells, the majority is subjected to the process of glycolysis to form pyruvate and generate ATP, NADH, and FADH2. The generated NADH and FADH2 are transported to the mitochondria from the cytosol by the malate-aspartate enzyme or by the glycerol phosphate shuttle where they are used as electron donors during oxidative phosphorylation. The electrons from NADH or FADH2 are transferred to the molecular oxygen (O_2_) in the mitochondrial respiratory chain complexes I–IV to generate ATP. During this process, the majority of O_2_ in normal physiological states is reduced to water and <1% is converted into O^2−^. However, when mitochondrial dysfunction exists, or in the state of persistent hyperglycemia, it produces an excessive leakage of electrons in complex I and in the interface between the coenzyme Q and complex III, since the mitochondrial respiratory chain is the primary source in the generation of O^2−^ in the cell. Therefore, the flow of O^2−^ outside the mitochondria is increased [24]. The protein complexes of the internal mitochondrial membrane are used to pump protons in the intermembranal space. This process is efficient, but a small percentage of the electrons can prematurely reduce the O_2_ forming O^2−^ producing OS in the mitochondria [25]. The mitochondria are highly dynamic and constantly experiencing the processes of fission and fusion. The fission gives, as a result, the production of strips or short mitochondrial spheres, and fusion promotes the production of a long filament [26]. Mitochondrial fission 1 protein (FIS1) is a key component in the mechanics of mitochondrial fission in the cells of mammals. In a previous study, mitochondrial fragmentation and the increase in expression of FIS1 in recently isolated venous endothelial cells in patients with DM were reported. In silencing the expression of FIS1, the dynamin-1-like protein (drp1) through a small ARN of interference increased the production of the ROS induced by hyperglycemia, which suggests that the increase in mitochondrial fission can affect the endothelial function through the increase in ROS [27]. The mitochondrial ROS play an important role in the complications of DM including DN [28]. The target cells, including the glomerular mesangial cells, the capillary endothelial cells of the retina, and the neuronal cells, are incapable of adequately regulating the concentrations of intracellular glucose in the diabetic ambiance [11]. Therefore, these cells are subjected to the extraordinary OS mediated by ROS [11].

### 1.5. Oxidative DNA Damage and Apoptosis

The persistent OS in the state of hyperglycemia leads to modifications of the DNA capable of producing damage to the mitochondrial genetic material (mtDNA) and the nuclear DNA (nDNA) [29]. The ROS, on inducing damage to the DNA, cause breakage of the single or double strands, altering the bases (histones) [30]. Because the mtDNA is deficient in histones, it could be more susceptible to oxidative damage. It has been demonstrated that the mtDNA damage is more extensive and persistent than the nDNA damage in human cells subjected to OS [31]. The persistent oxidative damage on mtDNA favors the appearance of mutations in the mitochondrial genome [32] giving way to mitochondrial dysfunction that increases the production of ROS and forms a vicious cycle in the mitochondria by producing intense oxidative damage capable of unchaining cell death by necrosis or apoptosis [33, 34]. The 8-hydroxy-2-deoxyguanosine (8-OHdG) is a marker of oxidative DNA damage that increases in the presence of OS. The 8-OHdG marker is a potentially mutagenic compound that participates as an inductor of apoptosis through the activation of the initiator and executor caspases and increases the expression of p53 proteins [35–37]. Previous studies show that the urinary levels of the 8-OHdG marker are found elevated in patients with DN compared to healthy subjects, suggesting systemic oxidative DNA damage in DN. As well, it has been previously demonstrated that the progression of DN is positively associated with the levels of excretion of the 8-OHdG marker [38]. A previous study, after follow-up for five years of patients with DN, reported a significant progression of DN with increased 8-OHdG levels compared to patients with lower levels of excretion of this marker [39].

**Figure 2:**
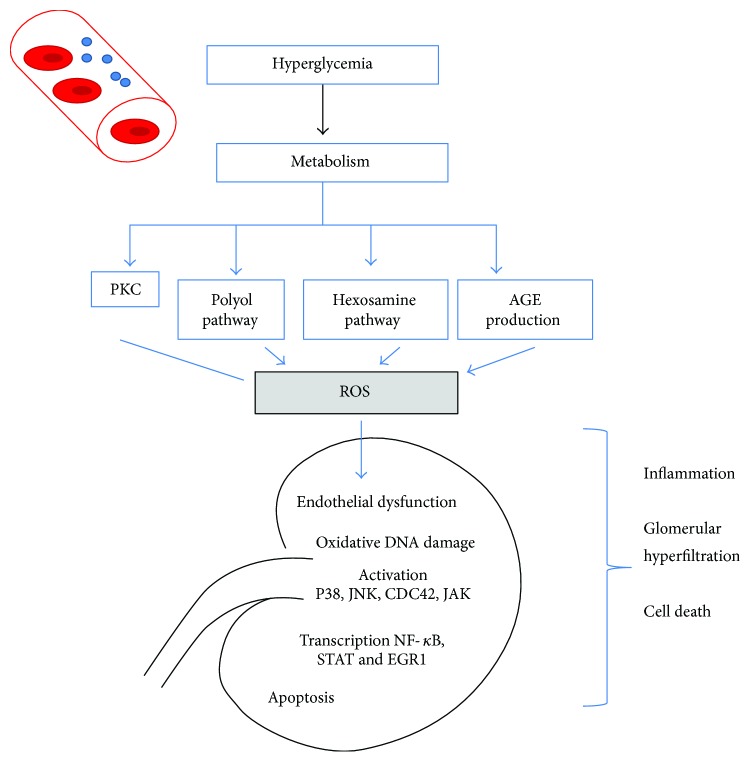
Hypothetical drawing of the apoptotic process in DN from hyperglycemia. Different signaling pathways that are activated in the state of hyperglycemia lead to OS and inflammation with capacity to carry out apoptosis in susceptible cells.

Apoptosis is the process of natural cell death, essential for the development and normal homeostasis of all multicellular organisms [40]. The state of hyperglycemia promotes apoptosis in various types of cells in DN, including the proximal tubule epithelial cells, without complete understanding of the mechanism [41]. Previously, it has been described that the apoptosis is caused by activation of a web of intracellular signaling pathways including the phosphoinositide 3-kinase (PI3K)/Akt signaling pathway [42]. However, it is known that the hyperglycemic environment induces apoptosis and contributes to the gradual loss of renal function in DN [43]. Apoptosis has been found in tubular epithelial cells, endothelial, and interstitial cells in renal biopsies in patients with DN [44] Figure 2.

### 1.6. Diabetic Nephropathy Current Treatment Strategies

There is no current treatment available to prevent the development of DN. The primary therapeutic strategies are based on the strict control of the primary, modifiable risk factors among which are the arterial hypertension, glucose levels, and dyslipidemia, although the control of these entities does not always prevent the progression of DN [45].

#### 1.6.1. Glycemia Control

The inadequate control of glycemia is the fundamental risk factor for the development and progression of DN. Consequently, it is imperative to avoid high levels of glucose [46]. Through previously published observational studies, the clear decrease in the incidence of DN in patients who were able to achieve better control of their glycemia is well known [47]. Strict glucose control reduces the risk of progression of severe albuminuria to the adequate GFT or the reduction of ESRD [48].

#### 1.6.2. Renin-Angiotensin-Aldosterone System

The renin-angiotensin-aldosterone system (RAAS) is an important pathway implicated in the pathogenesis and progression of DN [49]. The therapeutic blockage of the RAAS is achieved with the angiotensin-converting enzyme inhibitors (ACEi) or with the use of angiotensin II (Ang II) receptor blockers. Both strategies are effective in reducing proteinuria and the slow progression of DN or nondiabetic nephropathy by the hemodynamic/antihypertensive action, as well as by its anti-inflammatory/antifibrotic action. Ang II activates the NF-*κ*B and interacts with TGF-*β*. The anti-inflammatory action is produced through the inhibition of the NF-*κ*B-dependent pathways [50]. The RAAS inhibitors, including the ACEi and the ANG II receptor blockers, are both widely utilized to control arterial hypertension in diabetic patients. These medications are considered superior to other categories of antihypertensive medications in the treatment of DN due to their capacity to reduce the intraglomerular pressure and prevent proteinuria by favoring the dilation of the efferent glomerular arteriole. The grade of proteinuria in glomerular disease is directly related to the intraglomerular pressure. Thus, the reduction induced by the excretion of proteins causes a decrease in the intraglomerular pressure by improving renal function. The decrease in the excretion of proteins has a predictive significance for better or worse renal prognosis [51].

#### 1.6.3. Pentoxifylline

The pentoxifylline has important anti-inflammatory properties and offers beneficial effects on the microcirculatory blood flow due to its rheological properties. In patients with DM, it is associated with the reduction of urinary albumin excretion with possible beneficial effects on the GFR [52]. Pentoxifylline has also been demonstrated to reduce the urinary excretion of proteins in DM with normal renal function or with renal insufficiency [53]. The antiproteinuria effect has been related to reduction in concentrations of the tumor necrosis factor alpha (TNF-*α*), an important proinflammatory cytokine [54], inhibits the transcription of the TNF-*α* gene, and blocks the accumulation of mitochondrial ribonucleic acid (mtRNA), significantly reducing the levels of TNF-*α* and the urinary excretion of proteins without causing metabolic or hemodynamic changes [55]. The pentoxifylline has the capacity to modulate other cytokines and proinflammatory molecules (IFN, IL-10, and IL-6) on attenuating the processes of the inflammatory response (activation, adhesion, and phagocytosis) without causing metabolic or hemodynamic changes [56].

#### 1.6.4. Albumin

The albumin is continuously exposed to OS [57]. OS is associated with renal dysfunction in patients with kidney failure, and the plasma albumin is the object of massive oxidation [58]. The albumin has antioxidant properties and is the master antioxidant protein of the plasma. The structural stress induced by nonenzymatic glycation or the presence of ROS deteriorates the antioxidant capacity of the albumin. The deterioration of the antioxidant capacity of the albumin is a factor strongly associated with the development of complications in DM [59]. Evidence of protein stress has been demonstrated through the detection of the carbonyl content and of the dityrosine in patients with DN [60]. A new component of the antioxidant capacity of albumin was described, and it has to do with the intrinsic component denominated response surplus (RS). This component represents the antioxidant response that is produced when the proteins suffer structural disorder due to some stress factor (temperature, short-wave ultraviolet (UV) light, and ROS). The change in antioxidant capacity of the proteins is narrowly related to its molecular structure. The changes in molecular structure are clearly related to the passive redox state of thiol groups and particularly with the active thiol group of the albumin redox (Cys-34). The antioxidant capacity of any biological system is very complex, and the albumin dependent on Cys-34 represents a passive component while the RS system represents an active component related to the changes in molecular structure [61]. The antioxidant capacity of albumin decreases with the reduction of the GFR and the advancement of the stages of DN because of the oxidation of the thiol groups, derived essentially from Cys-34. In the oxidative state, the free thiol groups react, which results in the formation of progressively more oxidized species. The reversible formation of sulfinic and sulfenic acid maintains the redox state of the plasma to the moderate, unprolonged exposure to OS [62]. With higher levels of ROS, the prolonged exposure to OS produces the formation of sulfonic acid as an irreversible or end-product of the oxidation of the Cys-34-SH [63].

#### 1.6.5. Vitamins

Vitamin E suppresses albuminuria in patients with DM without cardiovascular disease preserving renal function [64]. Patients with oxidative stress due to low levels of genetically determined antioxidant haptoglobin, who received vitamin E, had significantly lower incidence of vascular events compared to healthy controls [65]. In a double-blind, placebo-controlled, crossover trial over 8 months, 36 subjects with type 1 DM and 9 subjects without diabetes were evaluated. The subjects ingested 1800 IU of vitamin E/day or placebo for 4 months and measured blood flow in the retina by fluorescein angiography and renal function by normalized creatinine clearance in urine samples. Treatment with vitamin E appears effective in normalizing hemodynamic abnormalities of the retina and improving renal function in patients with type 1 DM. The authors report no toxicity at the dose administered [66]. The administration of vitamin C alone or in combination with vitamin E has been recommended to decrease microalbuminuria. In a short study with small sample performed in type 1 DM patients with <10 years history of the illness who received a dose of 1800 IU/d of vitamin E, the authors demonstrated restoration of the renal function [65]. However, in the cardiac study (HOPE) with 4 years of follow-up where they evaluated the prevention in 3600 diabetic patients who received vitamin E supplements in a dose of 400 IU/day, they did not find a significant reduction in cardiovascular risk [67].

#### 1.6.6. Alpha-Lipoic Acid

Lipoic acid is a derivative of the short-chain fatty acid octanoic acid. The *α*-lipoic acid is a coenzyme produced endogenously that undertakes an essential role in the reactions of the mitochondrial dehydrogenase. The production source of lipoic acid is the intestinal medium. The lipoic acid bound to the food proteins and can be released by an intestinal amidase. Some bacteria encode an amidase called lipoamidasa capable of releasing lipoic acid from the intact 2-oxo acid dehydrogenase complexes. In fact, a lipoamidasa enzyme activity must be responsible for being free and available lipoic acid in nature [68]. The *α*-lipoic acid or its reduced form (dihydrolipoic acid) extinguishes various ROS in the lipid phase and in the aqueous phase on chelating transition metals and preventing lipid peroxidation of the membrane and protein damage through interactions with vitamin C and glutathione. The *α*-lipoic acid participates in the recycling of vitamins C and E by increasing the cellular levels of glutathione and suppressing the nonenzymatic glycation [69]. Treatment with *α*-lipoic acid reduces the markers of OS in the plasma of patients with DM with deficient control of glycemia [70]. Experimental studies in rats reported that the lipoic acid synthetase (Lias) deficiency induces manifested DN with production of microalbuminuria, thickening of the basal glomerular membrane, proliferation of the mesangial matrix, and hypertension compared to the diabetic controls without Lias deficiency [71]. Wang et al. treated diabetic rats with *α*-lipoic acid and demonstrated that in serum and renal cortex, the content of malondialdehyde (MDA), the activity of the superoxide dismutase (SOD), and the mitochondrial swelling were significantly reduced and the mitochondrial membrane potential significantly increased compared to the group of diabetic rats without the *α*-lipoic acid [72].

#### 1.6.7. Coenzyme Q10

The 2,3-dimethoxy-5-methyl-6-decaprenyl-benzoquinone is also called coenzyme Q10 or ubiquinone. The Q refers to the chemical quinone group and 10 to the number of chemical subunits of isoprene in the tail of the molecule. Coenzyme Q10 is present in mitochondria in the majority of the eukaryotic cells. Coenzyme Q10 is a vitamin-like substance; it is lipid soluble in nature and hydrophobic interior of the phospholipid bilayer of the cell membrane. Coenzyme Q10 exists in a wide range of dietary items including meat, fish, vegetable oils, and nuts and has a potent anti-inflammatory, antiulcer, antioxidant, and antidiabetic activity [73–76]. Coenzyme Q10 is an important component in the transport of electrons and participates in the cellular aerobic respiration generating energy in the ATP form. Ninety-five percent of the energy in the human body is generated by the mitochondria [77]. Coenzyme Q10 prevents alterations in function and mitochondrial morphology, glomerular hyperfiltration, and proteinuria in diabetic rats, emphasizing the role of the mitochondria in the pathogenesis of DN and the benefits in preventing the increase in OS [78]. However, its effect on the prevention of glomerulosclerosis is controversial [79]. An experimental study in diabetic rats treated with coenzyme Q10, metformin, or coenzyme Q10 + metformin showed significant decrease in MDA levels (*p* < 0.01, *p* < 0.05, and *p* < 0.001), respectively, versus diabetic rats not treated. Coenzyme Q10 or coenzyme Q10 + metformin showed significant increase in the activity of antioxidant enzymes SOD and catalase (*p* < 0.001) [80]. On the other hand, segmental focal glomerulosclerosis and collapsed glomerulopathy are common causes of nephrotic syndrome. The PDSS2 gene is required for the synthesis of the decaprenyl tail of coenzyme Q10 in humans. The deficiency manifests itself in the lymphoblastoid cell lines [81].

#### 1.6.8. Resveratrol

The resveratrol, 3,5,4′-trihydroxy-trans-stilbene, is a polyphenolic phytoalexin that is found in natural form in many plants such as grapes, berries, red wine, and legumes, presenting numerous health benefits [82]. The resveratrol is one of the most important natural stilbenes that has demonstrated having health promoter properties in possessing antioxidant, anti-inflammatory, cardioprotector, antidiabetic, anticancerous, chemoprotector, and neuroprotector effects [83]. Several studies once have informed on the potential health benefits with resveratrol in cardiac and kidney diseases [84]. The resveratrol has similar properties to insulin in DM. It has the capacity to protect the cells against OS in exhibiting concurrent anti- and proinflammatory effects. Resveratrol positively regulates the expression and activation of the AMP-activated protein kinase (AMPK), which can contribute to the beneficial effects in DN in the early stages [85].

#### 1.6.9. Inhibitors of the HMG-CoA Reductase

Inhibitors of the 3-hydroxy-3-methylglutaryl CoA (HMG-CoA) reductase or “statins” are potent inhibitors of the biosynthesis of cholesterol. Statins are very useful for the treatment of patients with dyslipidemia, although the effect of the statins in patients with DN is controversial. The HMG-CoA reductase inhibitors could be key medications in the presence of low-grade inflammation and endothelial dysfunction in DN, by reducing the proinflammatory pathways [86]. Clinical studies performed to look for the beneficial effect of statins on kidney function in DN are still controversial. In a subanalysis, it was discovered that the management with 10 mg and 80 mg of atorvastatin increases the glomerular filtration rate [87], while in the test of preventive intervention in kidney disease and ESRD (PREVEND-IT), the treatment with 40 mg of pravastatin did not improve the GFR [88]. In a recent meta-analysis, the authors published that statins significantly reduce albuminuria, the rate of excretion of urinary albumin, that the efficacy of renal function is dependent on the length of history of DM, and that the effect is better in type 2 DM with nephropathy [89].

#### 1.6.10. Inhibitors of COX-2 and Aspirin

The inhibitors of cyclooxygenase-2 (COX-2) are the primary anti-inflammatory agents. In a reported study, the authors suggested that the administration of aspirin could diminish albuminuria in patients with DN [90]. As well, it was reported that the combination of aspirin and AT1 receptor blockers decreased the progression of DN and inflammatory markers compared to the treatment with aspirin alone [91]. The COX-2 inhibitors could increase the renal hemodynamics by diminishing the expression of the profibrotic cytokines [92]. However, treatment with 200 mg/d of the COX-2 inhibitor for six weeks did not demonstrate a decrease in DN [93]. Therefore, the administration of COX-2 inhibitors for the treatment of DN continues to be controversial.

### 1.7. Diabetic Nephropathy Treatment Future Perspectives

#### 1.7.1. Nuclear Factor-Like 2 NFE2

The nuclear factor-like 2 NFE2 (Nrf2) is considered the master regulator of the response of cellular detoxification and of the redox state. Nrf2 provides protective action for diverse damages caused by OS [94]. After cellular exposure to OS, Nrf2 free of Kelch-like ECH-associated protein (KEAP1) translocates to the nucleus and binds to elements sensitive to antioxidants in the genes that code for antioxidant enzymes like the NADPH quinone dehydrogenase (NQO1), glutathione S-transferase, heme oxygenase-1 (HO1), and *γ*-glutamil-cysteine-synthetase, increasing its expression to undertake a role of detoxification as an antioxidant and anti-inflammatory effects [95]. Therefore, the positive regulation of the expression and function of the Nrf2 by different methods could provide preventative effects on the oxidative damage induced by DM in DN. The first study in which the activator Nrf2 was used to prevent the damage induced by hyperglycemia was done by Yang et al. These authors used sulforaphane (SNF) [1-isothiocyanato-4-(methylsulfinyl)-butane] [96]. Sulforaphane is an isothiocyanate of natural origin produced by cruciferous vegetables such as broccoli. Sulforaphane has the ability to induce the nuclear translocation of Nrf2 with significant increase of the antioxidant genes downstream, (expression > 3–5 times of transketolase and glutathione reductase) [97]. The treatment with SNF significantly impeded the hyperglycemia from increasing the formation of ROS and the activation of the hexosamine and PKC pathways [98].

#### 1.7.2. Adiponectin

The adiponectin is a hormone of 244 amino acids with a weight of 30 kDa produced by the adipocytes through the apM1 gene. The adiponectin structure is similar to the collagens VIII and X and the complement factor C1q. The hormone exists as a multimerer in the circulation of proteins of low, medium, and high molecular weights [99]. Two types of receptors have been reported for adiponectin (adipoR1 and adipoR2) in the skeletal muscle, liver, and endothelial cells. Its function includes the antiatherogenesis, anti-inflammation, and sensitization to insulin [100]. The adiopoR1 receptor mediates the increase in protein kinase activated by the adenosine monophosphate 5′ (AMPK). adiopR2 is capable of activating the receptor for the peroxisome proliferator-activated receptor alpha (PPAR*α*) [101]. It seems that the adiponectin of high molecular weight improves the sensitivity to insulin more than the adiponectin of low molecular weight [102]. Kacso et al. studied patients with type 2 DM over one year and found that when the levels of adiponectin were low, they predicted progression of the kidney disease characterized by increase in the albumin/creatinine relationship in urine [103]. Adiponectin is found in breast milk, fish intake, Mediterranean diet, coffee consumption, and omega 3 [104, 105].

#### 1.7.3. Inhibition of the NF-*κ*B

Several lines of evidence support the fundamental role of the transcriptional factor NF-*κ*B in the development of DN in the mesangial cells [106], in the glomerular endothelial cells [107], and in the podocytes [108]. Inhibition of the transcriptional factor NF-*κ*B in the kidney using the peroxisome (proliferator-activated receptor-*γ* (PPAR-*γ*)) has the ability to improve DN in animal models [109]. Pioglitazone regulates the phosphorylation of p66 (Shc) by integrating many signaling pathways that affect mitochondrial function, by reducing protein kinase C-beta, and the PPAR-*γ* not only improve the metabolic alterations of DM and DN, they also protect against nondiabetic CKD in experimental models and could benefit aging-related renal injury by improving mitochondrial function [110]. Nevertheless, there is no existing clear evidence on the efficacy of inhibiting the NF-*κ*B factor to slow the progression of DN [111]. The PPAR-*γ* and NF-*κ*B could signify an interesting therapeutic target.

#### 1.7.4. Rapamycin

The objective of the mammalian rapamycin (mTOR) is a serine/threonine-specific kinase that mediates cellular proliferation, survival, and size of the mass [112]. The rapamycin reduces the activity of mTOR that is augmented in the hyperglycemic state and mediates the renal changes in DN by favoring the mesangial proliferation [113]. The mTOR inhibitors are risk factors for DN because they cause hyperglycemia. Reports inform on their efficacy in the treatment of DN [114]. It should be considered that sirolimus inhibits the phosphorylation induced by the glucose of p70S6 kinase and its substrate, ribosomal protein S6 in mesangial cells. The inhibition of mTOR with sirolimus attenuates the morphogic and functional disturbances of the kidneys of diabetic patients, according to reports from a type 2 DM model in rats [115]. The rapamycin significantly reduces the accumulation of inflammatory cells, including monocytes and macrophages associated with the progression of DN, and it also reduces the liberation of proinflammatory cytokines and chemokines, including MCP-1, normal and secreted regulated T cells (RANTES), and IL-8 [116]. Thus, the administration of rapamycin is useful as an anti-inflammatory medication, which could suggest an attractive therapeutic treatment in the management of DN.

#### 1.7.5. Inhibition of PKC Activation

The hyperglycemic state and resistance to insulin induce the activation of the PKC. Activation of the PKC alters the molecules of cellular signaling, including the proinflammatory cytokines like the NF-*κ*B, IL-6, and TNF-*α* and plasminogen activator inhibitor-1 (PAI-1) in the vascular cells, including the endothelial and mesangial cells [117]. It has been shown that ruboxistaurin (RBX), a selective inhibitor of the PKC*β* isoform, prevents DN in rodent models through the inhibition of the accumulation of the ECM and the TGF-*β* by improving insulin signaling [118]. The *nuls* diabetic rats with PKC*β* demonstrated a decrease in albuminuria and mesangial proliferation [119]. In a phase II clinical trial, it was demonstrated that the treatment with RBX in DM significantly diminished albuminuria and maintained the estimated GFR (eGFR) stable. Hyperglycemia can activate the isoforms of PKC*β* that potentiate the toxic effect of Ang II in glomerular endothelial cells and decrease receptor 1 of the glucagon-like peptide (GLP-1), which leads to resistance to treatment with GLP-1 [120].

#### 1.7.6. Inhibitors of Sodium-Glucose Cotransporter-2 (SGLT-2)

The role of the kidney in glucose homeostasis has led to the development of sodium-glucose cotransporter-2 inhibitors (SGLT-2). SGLT-2 is a new class of antiglycemic drugs with the ability to reduce blood glucose by inhibiting sodium-glucose transporter in the proximal tubule of the kidney by improving renal excretion of glucose. Several of these inhibitors have been commercialized for treatment of hyperglycemia in patients with type 2 DM [121]. The treatment with SGLT2 has shown improvements in glycosylated hemoglobin (HbA1c), reduction in body weight, and moderate decrease in blood pressure [122]. SGLT2i has the ability to reduce albuminuria thereby reducing renal risk in the DN [51]. In patients with type 2 DM and stage 3 CKD, 100 mg/day of canagliflozin reduces albuminuria ~22% [123]. A study in patients with DM and hypertension who received renin blockers angiotensin aldosterone and dapagliflozin 10 mg/day showed ~35% reduction in albuminuria compared to placebo. The reduction was independent of changes in HbA1c, systolic blood pressure, body weight, or eGFR [124]. The decrease in serum uric acid is another nephroprotective mechanism of SGLT2i. The high levels of uric acid correlate highly with the risk of kidney damage and microvascular disease in DM [125]. Recently, it was announced that the administration of empagliflozine is associated with a significant reduction of the progression of kidney disease, including the rate of decrease of the eGFR, the progression of albuminuria, and the onset of renal replacement therapy [57]. The usefulness of SGLT-2 is still not well defined.

#### 1.7.7. Inhibitors of Dipeptidyl Peptidase 4 (DPP-4) and Glucagon-Like Peptide 1 (GLP-1)

The inhibitors of dipeptidyl peptidase 4 (DPP-4) are oral hypoglycemic that are useful in treating patients with type 2 DM. DPP-4 cleaves polypeptides from the amine terminal position, with a proline/alanine in the penultimate position. Therefore, the net physiological effect is a complex interaction between the resulting substrate and product profile in an environment of particular illness (instead of a specific signaling pathway). The cut substrates can activate or inactivate without having relevant function. The DPP-4 inhibitors reduce the levels of glucose in the blood increasing the half-life of the endogenous incretins like GLP-1 and the glucose-dependent insulinotropic polypeptide. The capacity of DPP-4 to cleave an additional quantity of substrate bound to the membrane that exercises nonenzymatic properties by the interaction or colocalization of other proteins/membrane receptors suggests that they could be a novel and stimulating therapeutic objective in DN [126].

## 2. Conclusion

Diabetic nephropathy is a microangiopathy that is prevalent in patients with type 1 and type 2 DM. The mechanism that unchains DN is still not entirely known. OS is a common and important factor that links hyperglycemia with the vascular complications through the metabolic changes to the molecules of the renal tissues and alterations in kidney hemodynamics. The persistent OS in the state of hyperglycemia favors oxidative DNA damage that is capable of producing damage to the genetic material mtDNA and to the nDNA upon favoring cell death by apoptosis. The kidney cells are predominantly susceptible to the hyperglycemic assault, resulting in greater flow of intracellular glucose and accelerating the oxidative phosphorylation of the mitochondria with the excessive leakage of O^2−^ and the significant decrease in the production of ATP. In patients with DN, it is convenient to stimulate the production of endogenous antioxidants or the administration of exogenous antioxidants that act as adjuvants to the management of the underlying pathology.

## Figures and Tables

**Table 1:** Classification of diabetic nephropathy [12].

Stage	Albuminuria (mg/g Cr) or proteinuria (g/g Cr)	GFR (mL/min/1.73 m^2^)
Stage 1 (prenephropathy)	Normoalbuminuria (<30)	≥30
Stage 2 (incipient nephropathy)	Microalbuminuria (30–299)	≥30
Stage 3 (overt nephropathy)	Macroalbuminuria (≥300) or persistent proteinuria (≥0.5)	≥30
Stage 4 (kidney failure)	Any albuminuria/proteinuria *status*	<30
Stage 5 (dialysis therapy)	Any *status* on continued dialysis therapy	

## References

[1] International Diabetes Federation Atlas of diabetes. http://www.diabetesatlas.org.

[2] World Health Organization Diabetes programme. http://www.who.int/diabetes/action_online/basics/en/index3.html.

[3] Muthuppalaniappan V. M., Sheaff M., Yaqoob M. M. (2015). Diabetic nephropathy. *Medicine*.

[4] Batuman V., Soman A. S., Schmidt R. J., Soman S. S. Diabetic nephropathy. https://emedicine.medscape.com/article/238946-overview.

[5] National Institute of Diabetes and Digestive and Kidney Diseases Diabetic kidney disease. https://www.niddk.nih.gov/health-information/diabetes/overview/preventing-problems/diabetic-kidney-disease.

[6] Federación Mexicana de Diabetes http://fmdiabetes.org/diabetes-México/.

[7] United States Renal Data System (USRDS) https://www.usrds.org/.

[8] Gross J. L., de Azevedo M. J., Silveiro S. P., Canani L. H., Caramori M. L., Zelmanovitz T. (2005). Diabetic nephropathy: diagnosis, prevention, and treatment. *Diabetes Care*.

[9] Grunwald J. E., Alexander J., Ying G. S. (2012). Retinopathy and chronic kidney disease in the Chronic Renal Insufficiency Cohort (CRIC) study. *Archives of Ophthalmology*.

[10] Martínez-Castelao A., Navarro-González J., Górriz J., de Alvaro F. (2015). The concept and the epidemiology of diabetic nephropathy have changed in recent years. *Journal of Clinical Medicine*.

[11] Mogensen C. E., Christensen C. K., Vittinghus E. (1983). The stages in diabetic renal disease: with emphasis on the stage of incipient diabetic nephropathy. *Diabetes*.

[12] Haneda M., Utsunomiya K., Koya D. (2015). A new classification of diabetic nephropathy 2014: a report from joint committee on diabetic nephropathy. *Clinical and Experimental Nephrology*.

[13] Rossing P. (2006). Diabetic nephropathy: worldwide epidemic and effects of current treatment on natural history. *Current Diabetes Reports*.

[14] Oates P. J. (2002). Polyol pathway and diabetic peripheral neuropathy. *International Review of Neurobiology*.

[15] Koya D., Jirousek M. R., Lin Y. W., Ishii H., Kuboki K., King G. L. (1997). Characterization of protein kinase C beta isoform activation on the gene expression of transforming growth factor-beta, extracellular matrix components, and prostanoids in the glomeruli of diabetic rats. *The Journal of Clinical Investigation*.

[16] Forbes J. M., Thallas V., Thomas M. C. (2003). The breakdown of preexisting advanced glycation end products is associated with reduced renal fibrosis in experimental diabetes. *The FASEB Journal*.

[17] Manda G., Checherita A. I., Comanescu M. V., Hinescu M. E. (2015). Redox signaling in diabetic nephropathy: hypertrophy versus death choices in mesangial cells and podocytes. *Mediators of Inflammation*.

[18] Lee H. B., Yu M. R., Yang Y., Jiang Z., Ha H. (2003). Reactive oxygen species-regulated signaling pathways in diabetic nephropathy. *Journal of the American Society of Nephrology*.

[19] Madeo F., Fröhlich E., Ligr M. (1999). Oxygen stress: a regulator of apoptosis in yeast. *The Journal of Cell Biology*.

[20] Forbes J. M., Coughlan M. T., Cooper M. E. (2008). Oxidative stress as a major culprit in kidney disease in diabetes. *Diabetes*.

[21] Duchen M. R. (2004). Roles of mitochondria in health and disease. *Diabetes*.

[22] Ceriello A., Morocutti A., Mercuri F. (2000). Defective intracellular antioxidant enzyme production in type 1 diabetic patients with nephropathy. *Diabetes*.

[23] Baynes J. W., Thorpe S. R. (1999). Role of oxidative stress in diabetic complications: a new perspective on an old paradigm. *Diabetes*.

[24] Addabbo F., Montagnani M., Goligorsky M. S. (2009). Mitochondria and reactive oxygen species. *Hypertension*.

[25] Voet D., Voet J. G., Pratt C. W. (2016). Fundamentals of biochemistry: life at the molecular level. *Chemistry & Biochemistry*.

[26] Westermann B. (2010). Mitochondrial fusion and fission in cell life and death. *Nature Reviews Molecular Cell Biology*.

[27] Shenouda S. M., Widlansky M. E., Chen K. (2011). Altered mitochondrial dynamics contributes to endothelial dysfunction in diabetes mellitus. *Circulation*.

[28] Hwang I., Lee J., Huh J. Y. (2012). Catalase deficiency accelerates diabetic renal injury through peroxisomal dysfunction. *Diabetes*.

[29] Fernyhough P., Huang T. J., Verkhratsky A. (2003). Mechanism of mitochondrial dysfunction in diabetic sensory neuropathy. *Journal of the Peripheral Nervous System*.

[30] Esposito L. A., Melov S., Panov A., Cottrell B. A., Wallace D. C. (1999). Mitochondrial disease in mouse results in increased oxidative stress. *Proceedings of the National Academy of Sciences of the United States of America*.

[31] Lieber M. R., Karanjawala Z. E. (2004). Ageing, repetitive genomes and DNA damage. *Nature Reviews Molecular Cell Biology*.

[32] Hollensworth S. B., Shen C. C., Sim J. E., Spitz D. R., Wilson G. L., LeDoux S. P. (2000). Glial cell type-specific responses to menadione-induced oxidative stress. *Free Radical Biology & Medicine*.

[33] Srinivasan S., Stevens M., Wiley J. W. (2000). Diabetic peripheral neuropathy: evidence for apoptosis and associated mitochondrial dysfunction. *Diabetes*.

[34] Ghafourifar P., Schenk U., Klein S. D., Richter C. (1999). Mitochondrial nitric-oxide synthase stimulation causes cytochrome*c* release from isolated mitochondria. Evidence for intramitochondrial peroxynitrite formation. *Journal of Biological Chemistry*.

[35] Grollman A. P., Moriya M. (1993). Mutagenesis by 8-oxoguanine: an enemy within. *Trends in Genetics*.

[36] Loft S., Vistisen K., Ewertz M., Tjønneland A., Overvad K., Poulsen H. E. (1992). Oxidative DNA damage estimated by 8-hydroxydeoxyguanosine excretion in humans: influence of smoking, gender and body mass index. *Carcinogenesis*.

[37] Lane D. P. (1992). p53, guardian of the genome. *Nature*.

[38] Xu G. W., Yao Q. H., Weng Q. F., Su B. L., Zhang X., Xiong J. H. (2004). Study of urinary 8-hydroxydeoxyguanosine as a biomarker of oxidative DNA damage in diabetic nephropathy patients. *Journal of Pharmaceutical and Biomedical Analysis*.

[39] Hinokio Y., Suzuki S., Hirai M., Suzuki C., Suzuki M., Toyota T. (2002). Urinary excretion of 8-oxo-7,8-dihydro-2′-deoxyguanosine as a predictor of the development of diabetic nephropathy. *Diabetologia*.

[40] Schwartzman R. A., Cidlowski J. A. (1993). Apoptosis: the biochemistry and molecular biology of programmed cell death. *Endocrine Reviews*.

[41] Allen D. A., Harwood S., Varagunam M., Raftery M. J., Yaqoob M. M. (2003). High glucose-induced oxidative stress causes apoptosis in proximal tubular epithelial cells and is mediated by multiple caspases. *The FASEB Journal*.

[42] Simone S., Gorin Y., Velagapudi C., Abboud H. E., Habib S. L. (2008). Mechanism of oxidative DNA damage in diabetes: tuberin inactivation and downregulation of DNA repair enzyme 8-oxo-7,8-dihydro-2′-deoxyguanosine-DNA glycosylase. *Diabetes*.

[43] Adeghate E. (2004). Molecular and cellular basis of the aetiology and management of diabetic cardiomyopathy: a short review. *Molecular and Cellular Biochemistry*.

[44] Droge W. (2002). Free radicals in the physiological control of cell function. *Physiological Reviews*.

[45] Hostetter T. H. (2001). Prevention of end-stage renal disease due to type 2 diabetes. *The New England Journal of Medicine*.

[46] de Boer I. H., Sibley S. D., Kestenbaum B. (2007). Central obesity, incident microalbuminuria, and change in creatinine clearance in the epidemiology of diabetes interventions and complications study. *Journal of the American Society of Nephrology*.

[47] Bojestig M., Arnqvist H. J., Hermansson G., Karlberg B. E., Ludvigsson J. (1994). Declining incidence of nephropathy in insulin-dependent diabetes mellitus. *The New England Journal of Medicine*.

[48] de Boer I. H., for the DCCT/EDIC Research Group (2014). Kidney disease and related findings in the diabetes control and complications trial/epidemiology of diabetes interventions and complications study. *Diabetes Care*.

[49] Ruggenenti P., Cravedi P., Remuzzi G. (2010). The RAAS in the pathogenesis and treatment of diabetic nephropathy. *Nature Reviews Nephrology*.

[50] Lee F. T., Cao Z., Long D. M. (2004). Interactions between angiotensin II and NF-*κ*B-dependent pathways in modulating macrophage infiltration in experimental diabetic nephropathy. *Journal of the American Society of Nephrology*.

[51] de Zeeuw D., Remuzzi G., Parving H. H. (2004). Proteinuria, a target for renoprotection in patients with type 2 diabetic nephropathy: lessons from RENAAL. *Kidney International*.

[52] Navarro J. F., Mora C. (1999). Antiproteinuric effect of pentoxifylline in patients with diabetic nephropathy. *Diabetes Care*.

[53] Navarro J. F., Mora C., Muros M., Macıéa M., Garcıéa J. (2003). Effects of pentoxifylline administration on urinary *N-*acetyl-*β-*glucosaminidase excretion in type 2 diabetic patients: a short-term, prospective, randomized study. *American Journal of Kidney Diseases*.

[54] Navarro J. F., Mora C., Muros M., García J. (2005). Additive antiproteinuric effect of pentoxifylline in patients with type 2 diabetes under angiotensin II receptor blockade: a short-term, randomized, controlled trial. *Journal of the American Society of Nephrology*.

[55] Doherty G. M., Jensen J. C., Alexander H. R., Buresh C. M., Norton J. A. (1991). Pentoxifylline suppression of tumor necrosis factor gene transcription. *Surgery*.

[56] Cooper A., Mikhail A., Lethbridge M. W., Kemeny D. M., Macdougall I. C. (2004). Pentoxifylline improves hemoglobin levels in patients with erythropoietin-resistant anemia in renal failure. *Journal of the American Society of Nephrology*.

[57] Kouoh F., Gressier B., Luyckx M. (1999). Antioxidant properties of albumin: effect on oxidative metabolism of human neutrophil granulocytes. *Il Farmaco*.

[58] Medina-Navarro R., Corona-Candelas I., Barajas-González S., Díaz-Flores M., Durán-Reyes G. (2014). Albumin antioxidant response to stress in diabetic nephropathy progression. *PLoS One*.

[59] Lim P. S., Cheng Y. M., Yang S. M. (2007). Impairments of the biological properties of serum albumin in patients on haemodialysis. *Nephrology*.

[60] Yamada N., Nakayama A., Kubota K., Kawakami A., Suzuki E. (2008). Structure and function changes of oxidized human serum albumin: physiological significance of the biomarker and importance of sampling conditions for accurate measurement. *Rinsho Byori*.

[61] Medina-Navarro R., Durán-Reyes G., Díaz-Flores M., Vilar-Rojas C. (2010). Protein antioxidant response to the stress and the relationship between molecular structure and antioxidant function. *PLoS One*.

[62] Lamprecht M., Greilberger J. F., Schwaberger G., Hofmann P., Oettl K. (2008). Single bouts of exercise affect albumin redox state and carbonyl groups on plasma protein of trained men in a workload-dependent manner. *Journal of Applied Physiology*.

[63] Turell L., Botti H., Carballal S. (2008). Reactivity of sulfenic acid in human serum albumin. *Biochemistry*.

[64] Gaede P., Poulsen H. E., Parving H. H., Pedersen O. (2001). Double-blind, randomised study of the effect of combined treatment with vitamin C and E on albuminuria in type 2 diabetic patients. *Diabetic Medicine*.

[65] Milman U., Blum S., Shapira C. (2008). Vitamin E supplementation reduces cardiovascular events in a subgroup of middle-aged individuals with both type 2 diabetes mellitus and the haptoglobin 2-2 genotype: a prospective double-blinded clinical trial. *Arteriosclerosis, Thrombosis, and Vascular Biology*.

[66] Bursell S. E., Clermont A. C., Aiello L. P. (1999). High-dose vitamin E supplementation normalizes retinal blood flow and creatinine clearance in patients with type 1 diabetes. *Diabetes Care*.

[67] Heart Outcomes Prevention Evaluation Study Investigators, Yusuf S., Dagenais G., Pogue J., Bosch J., Sleight P. (2000). Vitamin E supplementation and cardiovascular events in high-risk patients. The heart outcomes prevention evaluation study investigators. *The New England Journal of Medicine*.

[68] Cronan J. E. (2016). Assembly of lipoic acid on its cognate enzymes: an extraordinary and essential biosynthetic pathway. *Microbiology and Molecular Biology Reviews*.

[69] Packer L., Witt E. H., Tritschler H. J. (1995). Alpha-lipoic acid as a biological antioxidant. *Free Radical Biology & Medicine*.

[70] Borcea V., Nourooz-Zadeh J., Wolff S. P. (1999). *α*-Lipoic acid decreases oxidative stress even in diabetic patients with poor glycemic control and albuminuria. *Free Radical Biology & Medicine*.

[71] Yi X., Xu L., Hiller S. (2012). Reduced expression of lipoic acid synthase accelerates diabetic nephropathy. *Journal of the American Society of Nephrology*.

[72] Wang L., Wu C. G., Fang C. Q. (2013). The protective effect of *α*-lipoic acid on mitochondria in the kidney of diabetic rats. *International Journal of Clinical and Experimental Medicine*.

[73] Kamei M., Fujita T., Kanbe T. (1986). The distribution and content of ubiquinone in foods. *International Journal for Vitamin and Nutrition Research*.

[74] Schmelzer C., Lindner I., Rimbach G., Niklowitz P., Menke T., Döring F. (2008). Functions of coenzyme Q_10_ in inflammation and gene expression. *BioFactors*.

[75] Kohli Y., Suto Y., Kodama T. (1981). Effect of hypoxia on acetic acid ulcer of the stomach in rats with or without coenzyme Q10. *The Japanese Journal of Experimental Medicine*.

[76] Lenaz G., Fato R., Formiggini G., Genova M. L. (2007). The role of coenzyme Q in mitochondrial electron transport. *Mitochondrion*.

[77] Ernster L., Dallner G. (1995). Biochemical, physiological and medical aspects of ubiquinone function. *Biochimica et Biophysica Acta (BBA) - Molecular Basis of Disease*.

[78] Persson M. F., Franzén S., Catrina S. B. (2012). Coenzyme Q10 prevents GDP-sensitive mitochondrial uncoupling, glomerular hyperfiltration and proteinuria in kidneys from *db*/*db* mice as a model of type 2 diabetes. *Diabetologia*.

[79] Sourris K. C., Harcourt B. E., Tang P. H. (2012). Ubiquinone (coenzyme Q10) prevents renal mitochondrial dysfunction in an experimental model of type 2 diabetes. *Free Radical Biology & Medicine*.

[80] Maheshwari R. A., Balaraman R., Sen A. K., Seth A. K. (2014). Effect of coenzyme Q10 alone and its combination with metformin on streptozotocin-nicotinamide-induced diabetic nephropathy in rats. *Indian Journal of Pharmacology*.

[81] Gasser D. L., Winkler C. A., Peng M. (2013). Focal segmental glomerulosclerosis is associated with a *PDSS2* haplotype and, independently, with a decreased content of coenzyme Q_10_. *American Journal of Physiology-Renal Physiology*.

[82] Bertelli A. A. A., Das D. K. (2009). Grapes, wines, resveratrol, and heart health. *Journal of Cardiovascular Pharmacology*.

[83] Brasnyó P., Molnár G. A., Mohás M. (2011). Resveratrol improves insulin sensitivity, reduces oxidative stress and activates the Akt pathway in type 2 diabetic patients. *The British Journal of Nutrition*.

[84] Kondratyuk T. P., Park E. J., Marler L. E. (2011). Resveratrol derivatives as promising chemopreventive agents with improved potency and selectivity. *Molecular Nutrition & Food Research*.

[85] Chang C. C., Chang C. Y., Wu Y. T., Huang J. P., Yen T. H., Hung L. M. (2011). Resveratrol retards progression of diabetic nephropathy through modulations of oxidative stress, proinflammatory cytokines, and AMP-activated protein kinase. *Journal of Biomedical Science*.

[86] Ross R. (1999). Atherosclerosis — an inflammatory disease. *The New England Journal of Medicine*.

[87] Shepherd J., Kastelein J. J., Bittner V. (2008). Intensive lipid lowering with atorvastatin in patients with coronary heart disease and chronic kidney disease: the TNT (Treating to New Targets) study. *Journal of the American College of Cardiology*.

[88] Brouwers F. P., Asselbergs F. W., Hillege H. L. (2011). Long-term effects of fosinopril and pravastatin on cardiovascular events in subjects with microalbuminuria: ten years of follow-up of Prevention of Renal and Vascular End-stage Disease Intervention Trial (PREVEND IT). *American Heart Journal*.

[89] Shen X., Zhang Z., Zhang X. (2016). Efficacy of statins in patients with diabetic nephropathy: a meta-analysis of randomized controlled trials. *Lipids in Health and Disease*.

[90] Hopper A. H., Tindall H., Davies J. A. (1989). Administration of aspirin-dipyridamole reduces proteinuria in diabetic nephropathy. *Nephrology Dialysis Transplantation*.

[91] Mulay S. R., Gaikwad A. B., Tikoo K. (2010). Combination of aspirin with telmisartan suppresses the augmented TGF*β*/smad signaling during the development of streptozotocin-induced type I diabetic nephropathy. *Chemico-Biological Interactions*.

[92] Cheng H. F., Wang C. J., Moeckel G. W., Zhang M. Z., McKanna J. A., Harris R. C. (2002). Cyclooxygenase-2 inhibitor blocks expression of mediators of renal injury in a model of diabetes and hypertension. *Kidney International*.

[93] Sinsakul M., Sika M., Rodby R. (2007). A randomized trial of a 6-week course of celecoxib on proteinuria in diabetic kidney disease. *American Journal of Kidney Diseases*.

[94] Kobayashi M., Yamamoto M. (2006). Nrf2–Keap1 regulation of cellular defense mechanisms against electrophiles and reactive oxygen species. *Advances in Enzyme Regulation*.

[95] de Haan J. B. (2011). Nrf2 activators as attractive therapeutics for diabetic nephropathy. *Diabetes*.

[96] Yang L., Palliyaguru D. L., Kensler T. W. (2016). Frugal chemoprevention: targeting Nrf2 with foods rich in sulforaphane. *Seminars in Oncology*.

[97] Pall M. L., Levine S. (2015). Nrf2, a master regulator of detoxification and also antioxidant, anti-inflammatory and other cytoprotective mechanisms, is raised by health promoting factors. *Acta Physiologica Sinica*.

[98] Xue M., Qian Q., Adaikalakoteswari A., Rabbani N., Babaei-Jadidi R., Thornalley P. J. (2008). Activation of NF-E2–related factor-2 reverses biochemical dysfunction of endothelial cells induced by hyperglycemia linked to vascular disease. *Diabetes*.

[99] Adamczak M., Wiecek A. (2013). The adipose tissue as an endocrine organ. *Seminars in Nephrology*.

[100] Kadowaki T., Yamauchi T. (2005). Adiponectin and adiponectin receptors. *Endocrine Reviews*.

[101] Yamauchi T., Kadowaki T. (2008). Physiological and pathophysiological roles of adiponectin and adiponectin receptors in the integrated regulation of metabolic and cardiovascular diseases. *International Journal of Obesity*.

[102] Fisher F. F. M., Trujillo M. E., Hanif W. (2005). Serum high molecular weight complex of adiponectin correlates better with glucose tolerance than total serum adiponectin in Indo-Asian males. *Diabetologia*.

[103] Kacso I., Lenghel A., Bondor C. I. (2012). Low plasma adiponectin levels predict increased urinary albumin/creatinine ratio in type 2 diabetes patients. *International Urology and Nephrology*.

[104] Fields D. A., Schneider C. R., Pavela G. (2016). A narrative review of the associations between six bioactive components in breast milk and infant adiposity. *Obesity*.

[105] Fisman E. Z., Tenenbaum A. (2014). Adiponectin: a manifold therapeutic target for metabolic syndrome, diabetes, and coronary disease?. *Cardiovascular Diabetology*.

[106] Menini S., Amadio L., Oddi G. (2006). Deletion of p66^Shc^ longevity gene protects against experimental diabetic glomerulopathy by preventing diabetes-induced oxidative stress. *Diabetes*.

[107] Mima A., Hiraoka-Yamomoto J., Li Q. (2012). Protective effects of GLP-1 on glomerular endothelium and its inhibition by PKC*β* activation in diabetes. *Diabetes*.

[108] Mima A., Kitada M., Geraldes P. (2012). Glomerular VEGF resistance induced by PKC*δ*/SHP-1 activation and contribution to diabetic nephropathy. *The FASEB Journal*.

[109] Kamal F., Yanakieva-Georgieva N., Piao H., Morioka T., Oite T. (2010). Local delivery of angiotensin II receptor blockers into the kidney passively attenuates inflammatory reactions during the early phases of streptozotocin-induced diabetic nephropathy through inhibition of calpain activity. *Nephron*.

[110] Yang H. C., Deleuze S., Zuo Y., Potthoff S. A., Ma L. J., Fogo A. B. (2009). The PPAR*γ* agonist pioglitazone ameliorates aging-related progressive renal injury. *Journal of the American Society of Nephrology*.

[111] Wu J., Guan T. J., Zheng S. (2011). Inhibition of inflammation by pentosan polysulfate impedes the development and progression of severe diabetic nephropathy in aging C57B6 mice. *Laboratory Investigation*.

[112] Lieberthal W., Levine J. S. (2009). The role of the mammalian target of rapamycin (mTOR) in renal disease. *Journal of the American Society of Nephrology*.

[113] Nagai K., Matsubara T., Mima A. (2005). Gas6 induces Akt/mTOR-mediated mesangial hypertrophy in diabetic nephropathy. *Kidney International*.

[114] Velagapudi C., Bhandari B. S., Abboud-Werner S., Simone S., Abboud H. E., Habib S. L. (2011). The tuberin/mTOR pathway promotes apoptosis of tubular epithelial cells in diabetes. *Journal of the American Society of Nephrology*.

[115] Mori H., Inoki K., Masutani K. (2009). The mTOR pathway is highly activated in diabetic nephropathy and rapamycin has a strong therapeutic potential. *Biochemical and Biophysical Research Communications*.

[116] Yang Y., Wang J., Qin L. (2007). Rapamycin prevents early steps of the development of diabetic nephropathy in rats. *American Journal of Nephrology*.

[117] Yerneni K. K., Bai W., Khan B. V., Medford R. M., Natarajan R. (1999). Hyperglycemia-induced activation of nuclear transcription factor kappaB in vascular smooth muscle cells. *Diabetes*.

[118] Mima A., Ohshiro Y., Kitada M. (2011). Glomerular-specific protein kinase C-*β*-induced insulin receptor substrate-1 dysfunction and insulin resistance in rat models of diabetes and obesity. *Kidney International*.

[119] Ohshiro Y., Ma R. C., Yasuda Y. (2006). Reduction of diabetes-induced oxidative stress, fibrotic cytokine expression, and renal dysfunction in protein kinase C*β*–null mice. *Diabetes*.

[120] Gilbert R. E., Kim S. A., Tuttle K. R. (2007). Effect of ruboxistaurin on urinary transforming growth factor-*β* in patients with diabetic nephropathy and type 2 diabetes. *Diabetes Care*.

[121] Toto R. D. (2017). SGLT-2 inhibition: a potential new treatment for diabetic kidney disease?. *Nephron*.

[122] Zanoli L., Granata A., Lentini P. (2015). Sodium-glucose linked transporter-2 inhibitors in chronic kidney disease. *Scientific World Journal*.

[123] Yale J.-F., Bakris G., Cariou B. (2013). Efficacy and safety of canagliflozin in subjects with type 2 diabetes and chronic kidney disease. *Diabetes, Obesity & Metabolism*.

[124] Heerspink H. J. L., Johnsson E., Gause-Nilsson I., Cain V. A., Sjöström C. D. (2016). Dapagliflozin reduces albuminuria in patients with diabetes and hypertension receiving renin-angiotensin blockers. *Diabetes, Obesity & Metabolism*.

[125] Chonchol M., Shlipak M. G., Katz R. (2007). Relationship of uric acid with progression of kidney disease. *American Journal of Kidney Diseases*.

[126] Panchapakesan U., Pollock C. A. (2014). DPP-4 inhibitors—renoprotection in diabetic nephropathy?. *Diabetes*.

